# Lobomycosis in a Post-Covid 19 Patient: A Case Report and Review of Literature

**DOI:** 10.5146/tjpath.2023.01604

**Published:** 2023-09-15

**Authors:** Sateesh S Chavan, Thotadamane Nagaraja Chandrashekhar

**Affiliations:** Departments of Pathology, Karnataka Institute of Medical Sciences, Karnataka, India; Shivamogga Institute of Medical Sciences, Karnataka, India

**Keywords:** Lobomycosis, Lacaziosis, Chain of yeasts, Sequential budding, Connecting tubes

## Abstract

**Aim: **To document a case of lobomycosis and to discuss its epidemiology & diagnosis.

**Case Report: **A 53-year-old male presented with a history of nasal congestion, nasal discharge, and epistaxis following Covid 19 infection. On physical examination, there was necrotic slough in the nasal vestibule near the inferior turbinate. Scrapings and punch biopsy were taken from the lesion. Hematoxylin-eosin-stained sections showed necrotic and mucoid areas with mixed inflammatory cell infiltration and numerous budding yeasts 3- 7μm diameter in singles, and small clusters with single narrow based budding as well as multiple budding including sequential budding forming “chains of yeasts”. A diagnosis of Lobomycosis was made. Yeasts of lobomycosis are often confused with other yeasts such as *P. brasiliensis, Candida spp., B. dermatitidis, *and* Cryptococci,* but characteristic ‘sequential budding’ with a ‘chain of yeasts” aid in the final diagnosis. Demonstration of yeasts with characteristic chains either in tissue sections or in potassium hydroxide (KOH) preparation of scraped material, exudate, or exfoliative cytology is the mainstay in the diagnosis as the organisms are uncultivable *in vitro* in culture medium.

## INTRODUCTION

Lobomycosis is a chronic fungal infection caused by *Lacazia loboi,* a disease of humans and dolphins that is highly prevalent in tropical and coastal regions of Central and South America. The disease is often seen among forest workers, veterinarians, marine biologists, and those handling aquariums and is usually contracted following traumatic inoculation of organisms into subcutaneous tissue. Epidemiologically, the disease can be considered an occupational hazard as well as zoonotic. The most common presentation is ‘keloid like’ subcutaneous nodules (hence the name ‘keloidal blastomycosis’) on extremities and other exposed areas. The disease is diagnosed by demonstration of sequentially ‘budding yeasts’ forming a ‘chain of yeasts’ either in tissue sections or potassium hydroxide (KOH) preparation of exudate or exfoliative cytology of scrapings from lesions. The organism has not been isolated and grown* in vitro* in culture medium so far (1–3).

Here, we present a case of lobomycosis in a post-Covid 19 patient.

## CASE PRESENTATİON

A 53-year-old male carpenter by occupation presented with history of nasal congestion and nasal discharge, often with epistaxis. There was a history of recovery from Covid-19 infection. During his stay in quarantine with hospitalization, he had recurrent fever with maintained O2 saturation (ranged from 92% to 98%) and normal lung fields on high resolution computed tomography. He was on medication such as Azithromycin, Dexamethasone, Tamiflu, Ivermectin, Paracetamol, Zinc, and vitamin C. During the quarantine period, his laboratory investigations showed raised levels of C-reactive protein, D-dimer, random blood sugar (12.88mmol/L), ferritin levels, and lactate dehydrogenase levels.. He was a known diabetic and hypertensive, and was on short-term insulin therapy during the quarantine period. There was no travel history to tropical regions. He developed nasal congestion with discharge 4 weeks after recovery from the Covid-19 infection. Physical examination revealed a crusted ulcerative lesion with necrotic slough in the left nasal vestibule near the inferior turbinate. His routine investigations showed hemoglobin of 131gm/L, total leukocyte count of 9.3 x 109 cells/L with neutrophilia, platelet count of 210 x 109 cells/L, RBS-9.325mmol/L, ESR-40mm/1st hour, blood urea 1.887mmol/L and serum creatinine of 0.0611mmol/L

He underwent curettage and material was sent for fungal culture and histopathological examination. Fungal culture did not yield any growth.

**Figure 1:**
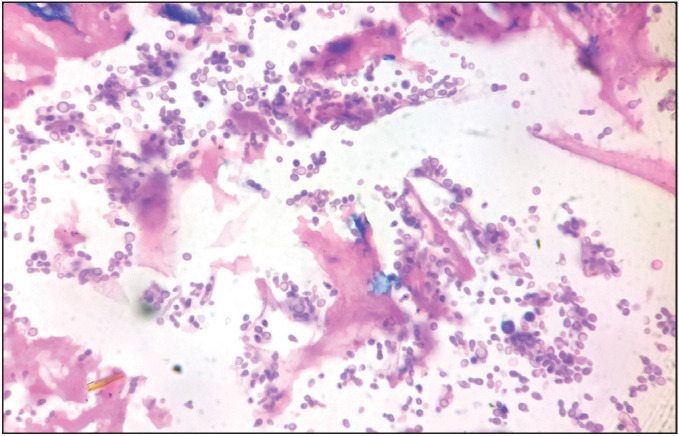
Numerous budding yeasts in mucoid & necrotic background [Haematoxylin & Eosin x 100].

**Figure 2:**
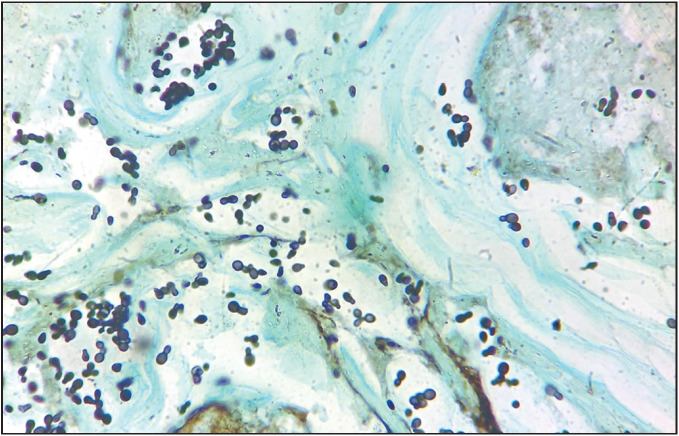
Numerous budding yeasts in mucoid & necrotic background [Gomori’s methenamine silver nitrate stain x 100].

**Figure 3:**
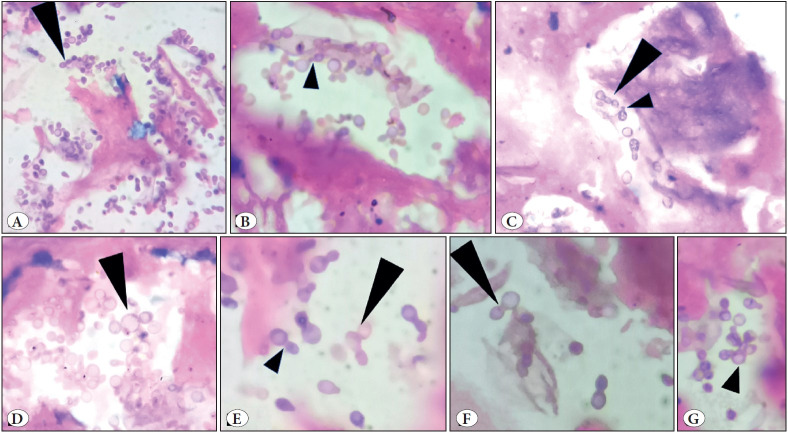
H & E. [Fig A, x 40; Fig B, C, D, x 100; Fig E, F, G x 200]. Numerous thick walled ‘narrow based’ budding yeasts in a mucoid to necrotic background, many of which show ‘tube like isthmus’ connecting parent cell with daughter cells [3E, short black arrow head]. Many of these yeasts show ‘retained tube’ after the detachment from parent cell [3B&C, short black arrow head]. They also showed characteristic multiple ‘sequential’ budding resulting in ‘chain of yeasts’ [3A, C, D &E, long black arrow heads] and multiple irregular ‘non sequential’ budding [3G, short black arrow head].

**Figure 4:**
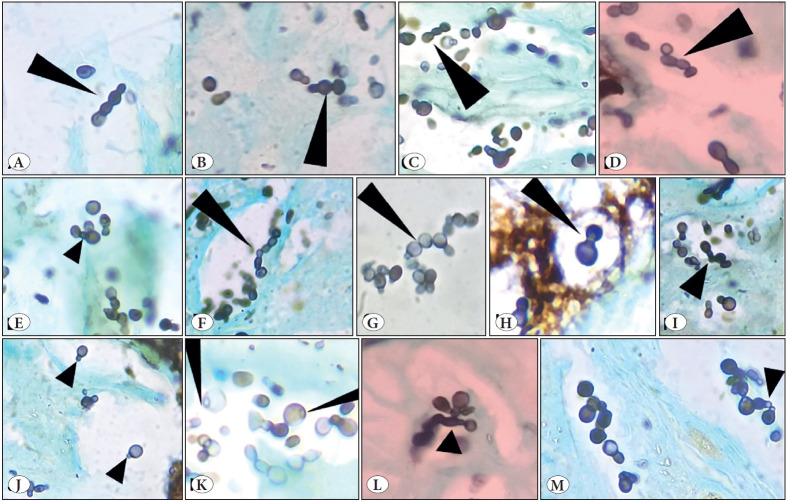
Gomori’s methenamine silver nitrate stain. [Fig A, B, C, E, F, G, I, J, L x 100; Fig D, H, K, M x 200]. Yeasts showed characteristic multiple ‘sequential’ budding resulting in ‘chain of yeasts’ [4A, B, C, D, F & G, long black arrow heads] and multiple irregular ‘non sequential’ budding [4E, I & L, short black arrow head]. Many of which show ‘tube-like isthmus’ connecting parent cell with daughter cells [4E, short black arrow head] and ‘retained tube’ after the detachment from parent cell [4J&M, short black arrow head]. They also showed characteristic ‘bud scars’ [4K, long black arrow heads

Formalin fixed paraffin embedded hematoxylin & eosin-stained sections showed necrotic and mucoid areas with mixed inflammatory cell infiltration including neutrophils, lymphocytes, and histiocytes (Figure 1). Amidst these are seen variably sized yeasts measuring 3- 7μm diameter with well delineated thick hyaline refractile wall, dispersed singly, in small clusters as well as in radiating chains. They showed ‘single’ *narrow based budding* (Figure 1, Figure 2) as well as ‘multiple’ narrow based budding including ‘*sequential budding’* resulting in “*chains of yeasts*” (Figure 3 and Figure 4) and multiple ‘*non sequential budding* (Figure 3 and Figure 4). Characteristically, the parent cell and daughter cells were connected by ‘narrow stalk’ or “*tube like isthmus*” (also called ‘*connecting tubes*’) (Figure 3 and Figure 4). Also, detached cells showed ‘*retained tube like structures*’ on one cell (Figure 3 and Figure 4) and *‘bud scars’* on the other (Figure 4). ‘*Tube like structures’* were also observed in yeasts with multiple ‘non sequential budding’ (Figure 4). Occasionally, budding yeasts showed “*hour glass appearance*” due to elongation of connecting tubes (Figure 4). The organisms were GMS (Gomori’s methenamine silver nitrate stain) positive (Figure 2, Figure 4). Histopathological diagnosis of lobomycosis was made.

The patient was given intravenous injection of broad-spectrum antifungal agent ‘liposomal Amphotericin B’ (1mg/kg) for the first 5 days and continued on days 14 and 21. In addition he was administered a combination of Itraconazole (100mg/day) and Clofazimine (100mg) daily for 6 months. After 9 months of follow up, the patient was free from recurrence. The patient was out of further follow-up.

## DISCUSSION

Lobomycosis, an emerging and enigmatic tropical disease, is a self-limited chronic subcutaneous mycosis also called ‘*Jorge Lobo Blastomycosis,’ keloidal blastomycosis, pseudo lepromatous blastomycosis, Caiabi leprosy, *and *Lacaziosis*. It was first described by the dermatologist Jorge Lobo in 1931 in a middle-aged forest worker (1,2). It is a disease of dolphins & humans, and to date, around 500 cases have been reported so far. Most cases occurred in the regions of the Amazon basin of South America and Central America in which climatic characteristics of tropical regions are suitable for organisms, making it the region the with highest number documented cases. The rest occurred in coastal regions of the United States, Europe, and South Africa and very few cases are reported in Asia including India (1,2). The disease can be considered an occupational hazard (forest workers, veterinarians, marine biologists) or it can be zoonotic particularly amongst aquarium workers who deal with rescue operations of dolphins (1–17).

It is caused by the fungi ‘*Lacazia loboi*.’ The fungus belongs to the order *Onyganeles*, family *Ajellomycetes*, genus *Paracoccidioides*, and species *Lacazia loboi*. The fungus has not yet been cultured and isolated so far. Because of the difficulty in culture and isolation of the agent, taxonomic nomenclature was difficult and challenging, and hence it was variably called *Glenosporella loboi*, *Blastomyces brasiliensis*, *Glenosporosis amazonica, Paracoccidioides loboi, Blastomyces loboi*, and *Lobomyces loboi*. However, these terms were considered invalid and ‘*Lacazia loboi*’ has been proposed and followed (4,7,9,10).

Though some characteristics of *Paracoccidioides brasiliensis *and* Lacazia loboi’* put them in the same taxonomic complex, they are phylogenetically distinct (3). Despite many attempts to isolate the organism by inoculations of animals including mice, armadillo, and turtles, they were unsuccessful (1–3,10). The fungus is saprophytic and probably hydrophilic. Although soil and vegetation appear to be the natural habitat, many cases are reported in marine and aquatic environment indicating that *L. loboi* may be a hydrophilic microorganism (3–5).

The lesions of lobomycosis are usually limited to the epidermis and dermis and rarely involve subcutaneous tissue. They are pleomorphic ranging from macule, papule, ulcerative, nodular sclerodermiform, and verrucoid. Most cases present with freely mobile subcutaneous, painless, pruritic nodules over exposed areas like the face, ears and extremities. They grow over a period of weeks or months to form large verrucoid or ulcerative nodules, often with satellite nodules (1,2,4,11).

Often there is traumatic inoculation of the organism into the subepithelial zone where dermal macrophages phagocytose them followed by proliferation of yeasts. These phagocytes and activated Th2 lymphocytes secrete anti-inflammatory cytokines including transforming growth factor β1(TGF β1) and IL-10. TGFβ1 inhibits the phagocytic activity of macrophages, and inhibits Nitric oxide and IFN-ϒ expression, creating a localized immunodeficient state and finally stimulates the process of fibrosis. Once in the subepithelial zone, the yeasts can access the lymphatics and can disseminate to other sites. The ‘satellite lesions’ observed in lobomycosis are often due to autoinoculation, and less commonly due to lymphatic spread. Clinically and pathophysiologically, the disease resembles *lepromatous leprosy *and* cutaneous leishmaniasis*, all of them presenting as an *antigen specific immunodeficient state *(4,12).

In the present case, the patient presented with a friable to firm crusted and ulcerated lesion covered with slough over the left nasal vestibule associated with nasal congestion and mucoid discharge from the maxillary sinus. Though in most cases there is no predisposing factor, in conditions of altered immunity the fungi proliferate and lesions tend to progress and may disseminate (1–5). In the present case, the patient was a known diabetic and recently recovered from Covid 19 infection, which might have contributed to altered immunity.

Since the organism is uncultivable, the diagnosis of lobomycosis is based on demonstrating the thick-walled hyaline spherical yeasts either in exudates using KOH (potassium hydroxide) preparation or in exfoliative cytology of the ulcerative lesion or in tissue sections with hematoxylin & eosin stain. However, histopathology is the ‘gold standard’ for establishing the diagnosis. Special staining techniques such as Gomori’s methenamine silver nitrate stain, Periodic acid Schiff stain, or Calcofluor stain can be used to readily demonstrate yeasts (1,2,4,5,18,19).

In Hematoxylin & Eosin stained tissue sections, the yeasts are faintly stained, and are ‘doubly refractile’, birefringent thick-walled hyaline round to oval ‘lemon shaped,’ 3- 7μm in diameter, with amphophilic to slightly basophilic homogeneous cytoplasm. The yeasts usually reproduce by single narrow based budding with **‘** ***tube-like structures’ or ‘isthmus’ *** connecting one another and typically they show formation of **‘** ***chains of 2- 5 yeasts’ *** due to** ‘** ***sequential secondary budding’*** from daughter cells. When budding is ‘multiple’ occurring on different points on the same cell with subsequent ‘secondary sequential budding’ from daughter cells, this results in the formation of **‘** ***radiating chains of yeasts’ *** (2). Characteristically one of the budded cells (usually daughter cells) show ‘***bud scars’*** and the other (usually parent cell) show ‘***retained tube-like structure.’*** Some yeasts also exhibit elongation of ‘connecting tubes’ resulting in ‘hour glass’ appearance (*hour glass yeasts*). Although pseudo-hyphae formation is rare, this elongation of connecting tubes may represent pseudo hyphae formation (1,2,4,5,18,19). In the present case, the organisms with their characteristic morphology including ‘chains of yeasts,’ bud scars, and tube-like structures were easily recognized in GMS stain.

The tissue response is variable from case to case, ranging from granulomatous inflammation in the dermis with hyperplasia of the overlying epidermis to suppuration with necrosis. Though the tissue response is variable from case to case, tinctorial morphology of the fungi are remarkably similar in all cases. Yeasts, once they enter the tissue, elicit either an ‘antigen specific granulomatous response’ rich in granulomas, multinucleated giant cells and with absent to minimal necrosis (most common) or create an ‘antigen specific immunodeficient state’ with necrosis and suppuration rich in neutrophils, lymphocytes, histiocytes and minimal to absent granulomas (less common). Typically, the dermis shows granulomas comprising epithelioid histiocytes and multinucleated giant cells including Langhans giant cells. The stromal fibrosis is also variable, usually minimal, and shows a narrow delicate network of reticulin fibers that surround and may extend into granulomas. They are well demonstrated by Masson-Trichrome stain. It is unusual to see suppuration and necrosis in lobomycotic lesions. The present case showed abundant degenerated mucoid and fibrinoid necrotic material with mixed inflammatory cells including neutrophils and histiocytes. Fibrosis and well-defined epithelioid cell granulomas were absent.

Lobomycosis is confused with other yeasts or yeast like organisms that show budding such as* P. brasiliensis, Candida, Cryptococci, *and* B. dermatitidis.* *Paracoccidioides brasiliensis,* which also reproduce by single and multiple budding, may be confused with lobomycosis*. *However,* P. brasiliensis *do not form ‘chains of cells ‘with ‘connecting tubes’ (which are typical of lobomycosis). The daughter cells of *P. brasiliensis* are usually smaller in size than the parent cell, whereas the daughter cells of lobomycosis usually are larger or are of the same size as the parent cell (1,2,4,5). Budding yeasts of Candida may also be confused with that of Lobomycosis but absence of pseudohyphae and presence of typical ‘chain of yeasts’ and ‘tube like isthmus’ will aid in diagnosis of the latter. Occasionally, some *Cryptococci *show multiple budding. The typical ‘chain of yeasts’ is absent. One can see with careful search the typical’ mucoid’ capsule (that is absent in lobomycosis). Infrequently, *Blastomyces dermatitidis* also show multiple budding but typically they are ‘*broad-based’* and they do not form a ‘chain of yeasts.’ The cell walls of *B. dermatitidis* are thick and doubly refractile (1,2,4,5). Further *P. brasiliensis, Candida, Cryptococci, *and* B. dermatitidis *can be grown in vitro in culture and isolated but not lobomycosis.

The mainstay of treatment is surgical excision or curettage or cryosurgery for recurrent cases. None of the antifungals proved effective. Itraconazole has shown partial response; hence it is often used with cryosurgery for recurrent cases. The newer azoles such as Posaconazole used along with wide local excision have proven effective with no remission (1,2,4,5). The present case was treated with combined Itraconazole and Clofazimine along with intravenous Amphotericin B, and showed no recurrence in the follow up period of 9 months. It is also observed that some cases with concurrent leprosy (particularly in endemic areas such as Amazon basin regions) responded well to multi drug therapy with Clofazimine and Rifampicin in combination with newer azoles (4). In the present case, the patient was also on Clofazimine in addition to Itraconazole and Amphotericin B, and responded well with no recurrence in the short term follow up period.

In conclusion*, Lobomycosis* is highly prevalent in tropical regions and coastal regions of Central and South America, but can be seen sporadically all over the world where there is tropical climate. Histopathology is the mainstay in diagnosis as the organism is uncultivable *in vitro* in culture medium. Demonstration of budding yeasts with characteristic ‘sequential budding’ resulting in ‘chains of yeasts’ is an important finding which aids in the diagnosis. Identification of the organism is important as none of the antifungal drugs are effective and the mainstay in treatment is wide surgical excision combined with newer azoles.

## Conflict of Interest

The authors declare that there is no conflict of interest.
